# Spread of Dual-Resistant *Mycoplasma genitalium* Clone among Men, France, 2021–2022

**DOI:** 10.3201/eid3104.241602

**Published:** 2025-04

**Authors:** Sabine Pereyre, C. Laurier-Nadalié, Carla Balcon, Nadège Hénin, Amandine Dolzy, Marie Gardette, Jennifer Guiraud, Cécile Bébéar, investigator group

**Affiliations:** University of Bordeaux–National Centre for Scientific Research, Bordeaux, France (S. Pereyre, N. Henin, C. Bébéar); Bordeaux University Hospital, Bordeaux (S. Pereyre, C. Laurier-Nadalié, C. Balcon, A. Dolzy, M. Gardette, J. Guiraud, C. Bébéar)

**Keywords:** *Mycoplasma genitalium*, bacteria, antimicrobial resistance, sexually transmitted infections, substitution A2058T, *mgpB* type, clonal spread, France

## Abstract

The 2058T macrolide resistance–associated mutation in 23S rRNA has emerged in *Mycoplasma genitalium* in France. Using *mgpB* typing, we documented the spread of a macrolide- and moxifloxacin-resistant ST159 clone, harboring the A2058T and ParC Ser83Ile mutations. In France, that clone is likely circulating among men who have sex with men.

Macrolide resistance in *Mycoplasma genitalium* is rapidly increasing worldwide (*1*). Reports have noted the spread of macrolide resistance in this species to be polyclonal (*2*–*4*), but a few resistant clones may be circulating, particularly in men (*3*). To search for the potential spread of *M*. *genitalium* macrolide-resistant clones in men in France, we performed a prospective nationwide survey to investigate the *mgpB* type of macrolide-resistant strains.

During September 15–October 15 in 2021 and 2022, we carried out a systematic collection of *M. genitalium*–positive specimens from men from diagnostic laboratories in France and detected resistance-associated mutations using 23S rRNA and *parC* gene Sanger sequencing (*5*). In cases of Sanger sequencing failure, our investigator group used the commercial kits ResistancePlus MG assay (SpeeDx, https://plexpcr.com/home-us) and MGMO qPCR (NYtor, https://www.nytor.nl; which detects 4 *parC* mutations associated with fluoroquinolone resistance) to increase the sensitivity of mutation detection, but those kits did not specify the mutation location. In a related investigation, members of our research team searched mutations in the quinolone resistance–determining region of GryA by Sanger sequencing among ParC-mutated strains (*3*). We performed typing on all macrolide-resistant strains using single nucleotide polymorphism analysis of the *mgpB* adhesin gene (*3*).

In this study, we collected *M. genitalium*–positive specimens from 229 male patients from 38 laboratories in France in 2021 and from 191 male patients from 37 laboratories in 2022. The overall prevalence of macrolide-resistant mutations was 53.2% (95% CI 47.9%–58.3%), and the percentage of fluoroquinolone resistant mutations was 25.2% (95% CI 20.8%–30.1%).

The most frequent macrolide resistance–associated mutation was A2059G (46.6%, *Escherichia coli* numbering), followed by the A2058T substitution (33.5%) and the A2058G mutation (19.5%). The proportion of A2058T transversion increased significantly in 2022 compared with data from 2018 (18.8% in 2018 vs. 33.5% in 2021–2022, p = 0.01) (*5*). Strains with the A2058T substitution harbored significantly more fluoroquinolone resistance–associated Ser83Ile substitutions in ParC (66.0%, *M*. *genitalium* numbering) than those harboring other macrolide resistance–associated mutations (14.6%; p<0.001) and the 23S rRNA wild-type strains (4.3%; p<0.001) (Table). The A20258T substitution was more frequently detected in men who have sex with men (MSM) (86.7%) and in the Paris area (49.1%) (Table).

**Table:** Characteristics of the 327 *Mycoplasma genitalium–*positive men with successful determination of the 23S rRNA sequence from a study investigating the spread of a dual-resistant *Mycoplasma genitalium* clone among men, France, 2021–2022*

Characteristic	A2058T	Other defined 23S rRNA macrolide resistance–associated mutations	Wild type	p value†
No. patients	55	109	163	
Age				
Mean age, y	35.0	34.3	33.2	NS
Median age, y	33.4	31.5	30.1	
Range, y	(18–68)	(19–71)	(2 mo–67 y)	
Reason for *M. genitalium* detection				
Urogenital symptoms	11 (34.4)	23 (40.4)	37 (44.6)	NS
No urogenital symptoms‡	21 (65.6)	34 (59.6)	46 (55.4)	
Unknown	23	52	80	
Sexual behavior in men				
MSM or bisexual men	26 (86.7)	31 (77.5)	34 (50.0)	<0.05§
MSW	4 (13.3)	9 (22.5)	34 (50.0)	
Unknown	25	69	95	
HIV status				
Positive	7 (22.6)	8 (12.1)	11 (12.8)	NS
Negative	24 (77.4)	58 (87.9)	75 (87.2)	
Unknown	24	43	77	
Geographic origin				
Paris area	27 (49.1)	34 (31.2)	58 (35.6)	<0.05¶
Other regions of France	28 (50.9)	75 (68.8)	105 (64.4)	
ParC QRDR mutations (*M. genitalium* numbering)				
None	12 (22.6)	83 (80.6)	129 (91.5)	<0.05
Ser83 Ileu	35 (66.0)	15 (14.6)	6 (4.3)	<0.05
Other ParC mutations associated with FQ resistance (Asp87Asn, Asp87Tyr, or Gly81Cys)	4 (7.5)	4 (3.9)	5 (3.5)	NS
Other undefined ParC mutations associated with FQ resistance#	2 (3.8)	1(0.9)	1(0.7)	NS
Undetermined	2	6	22	
GyrA QRDR mutations (*M. genitalium* numbering)**				
Mutations in Asp99 (Asp99Asn or Asp99Gly)	4 (10.8)	0	0	NS
Other GyrA mutations with unknown significance	0	2 (10.0)	0	
Wild type	33 (89.2)	18 (90.0)	7 (100.0)	
Undetermined	3	0	5	
*Chlamydia trachomatis* coinfection	4 (8.7)	4 (4.8)	18 (13.4)	<0.05††
*Neisseria gonorrhoeae* coinfection	5 (10.9)	5 (6.0)	7 (5.2)	NS

*Data are no. (%), except as indicated. A2058T is numbered according to *Escherichia coli* numbering. FQ, fluoroquinolone; MSM, men who have sex with men; MSW, men who have sex with women; NS, not significant; QRDR, quinolone resistance–determining region. †p value calculated by using the χ2 or Fisher test, as appropriate. ‡Sexually transmitted infection screening, HIV follow-up, or pre-exposure prophylaxis for HIV follow-up. §p value calculated between the numbers for A2058T and wild type, and between other defined 23S rRNA macrolide resistance–associated mutations and wild type. ¶p value calculated between numbers for A2058T and other defined 23S rRNA macrolide resistance–associated mutations and wild type. #Fluoroquinolone resistance–associated mutations were detected using the MGMO quantitative PCR kit (NYtor, https://www.nytor.nl), which does not specify the nature or location of the mutation. **GyrA mutations were only searched in the ParC-mutated strains. ††p value calculated between the numbers for other defined 23S rRNA macrolide resistance–associated mutations and wild type.

*mgpB* typing of macrolide-resistant specimens revealed 47 distinct sequence types (STs), including 16 new types numbered ST339, ST347, ST350–ST356, and ST359–ST365 (Figure). ST159 was the most frequent ST, representing 20.2% (33 of 163 specimens with successful *mgpB* typing among the 185 macrolide-resistant specimens). All ST159 strains for which the nature of the mutation could be determined harbored both the A2058T transversion in 23S rRNA and the Ser83Ile substitution in ParC (Figure). Moreover, 94.1% of ST159 were from MSM or bisexual persons, and 63.6% (21/33) were from the Paris region. All 5 strains harboring a substitution at position Asp99 (*M. genitalium* numbering) in GyrA—which is a position where mutations increase the likelihood of moxifloxacin failure (*6*)—were ST159, of which 4 harbored an A2058T transversion in 23S rRNA and 1 had an undefined 23S rRNA mutation (Table; Figure).

**Figure:**
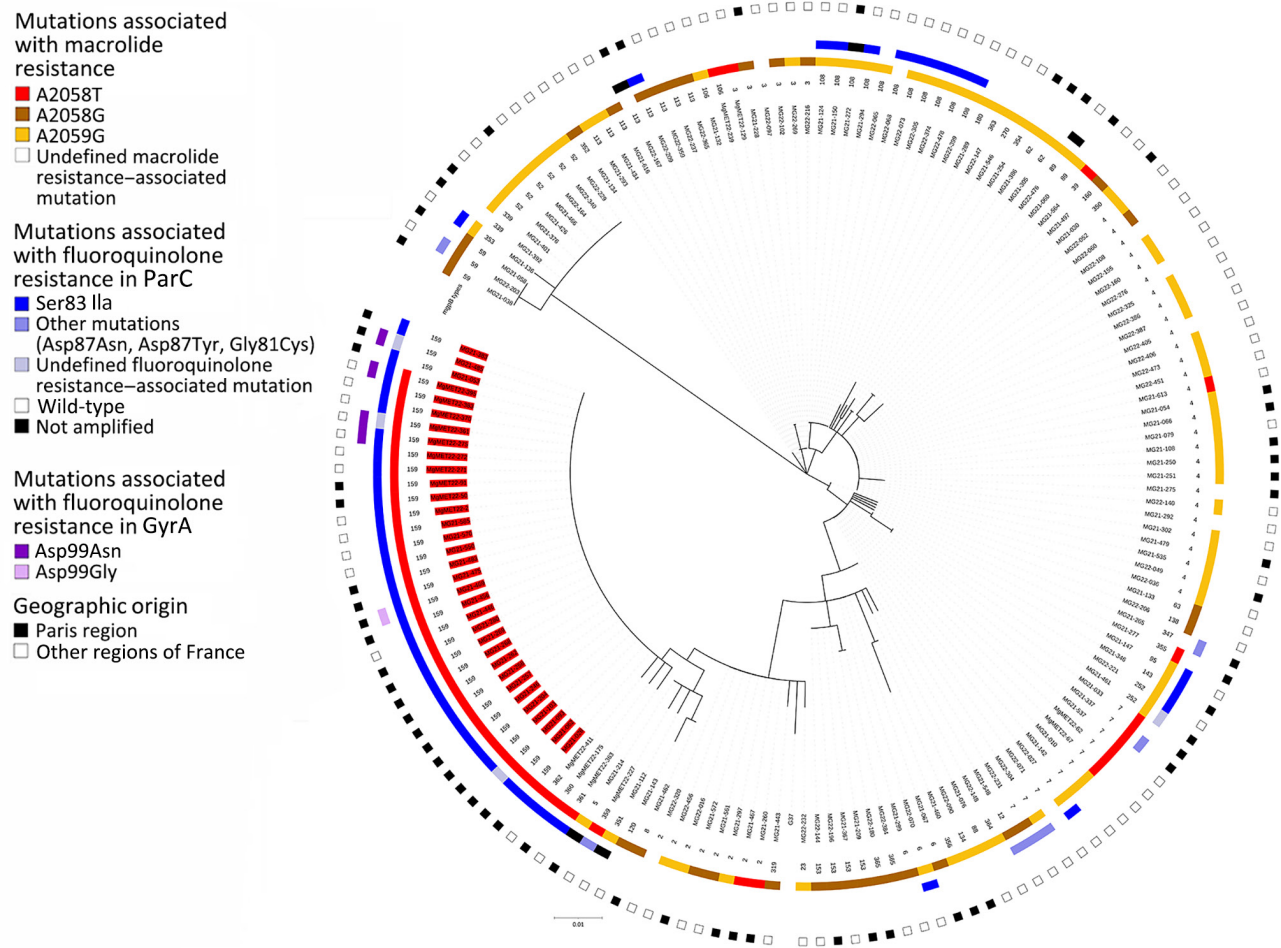
Maximum-likelihood tree in a study of spread of dual-resistant *Mycoplasma genitalium* clone among men, France, 2021–2022. The tree is based on the *mgpB* type of 163 successfully *mgpB*-typed *M.* *genitalium–*positive specimens harboring macrolide resistance–associated mutations. The tree was inferred using the maximum-likelihood method based on the Tamura 3–parameter model (T92 general plus invariable site model) constructed in MEGA7 (https://www.megasoftware.net). Branch support values were generated from 100 bootstrap replicates. The *M*. *genitalium* G37 strain (American Type Culture Collection no. 33530, https://www.atcc.org) sequence was used as a reference. The phylogenetic tree was annotated from the center to the periphery with the specimen name, the *mgpB* type, the macrolide resistance–associated mutation (*Escherichia* *coli* numbering), the fluoroquinolone resistance–associated *ParC* and *GyrA* mutations (*M*. *genitalium* numbering), and the geographic origin of the patient. Specimen names that were *mgpB* ST159 are highlighted in red. The phylogenetic tree was displayed and annotated using iTOL version 6 (https://itol.embl.de). Scale bar indicates the branch length corresponding to a genetic distance of 0.01, which indicates the average number of nucleotide substitutions per site.

Two studies have reported an increased prevalence of the A2058T mutation, one in the Netherlands and the other in Belgium, but those studies did not perform typing (*7*,*8*). In Spain, the emergence of the A20258T substitution has contributed to increasing macrolide resistance (*9*). A clonal spread was not retained by the authors despite a lower genetic diversity than expected. During 2018–2019, 8 MSM who lived in Paris were infected by a macrolide-resistant ST159 strain harboring a Ser83Ile ParC mutation, but the nature of the 23S rRNA mutation was not determined (*3*). Considering those data, we typed all the *M*. *genitalium* strains harboring the A2058T substitution detected in our previous 2018–2020 study (*5*). In total, 82.4% (28/34) A2058T strains belonged to ST159, suggesting that the A2058T-ST159 clone may have been spreading undetected for several years.

The limitations of this study were that 41.7% of the HIV and 55.2% of the sexual behavior data were missing, and only specimens harboring macrolide-resistant isolates were *mgpB*-typed. However, we previously showed that ST159 was much more prevalent among macrolide-resistant strains (10.6%) than among susceptible strains (0.2%) and was particularly prevalent in MSM (*3*). Thus, we strongly suspect the diffusion of a dual-resistant *M*. *genitalium* clone. Our research suggests that macrolide resistance detection kits would benefit from displaying the A2058T mutation in 23S rRNA, which is predictive of the fluoroquinolone resistance–associated mutation and thus of the possible failure of the first- and second-line treatments for *M*. *genitalium* infections. Considering the few therapeutic options in cases of macrolide and moxifloxacin resistance (*10*), the spread of such a dual-resistant clone is of concern.

In conclusion, we report an increase in the proportion of the A2058T mutation among macrolide-resistant *M*. *genitalium* strains in France during 2021–2022. We also note the spread of a dual-resistant 23S rRNA A2058T-ParC Ser83Ile *M*. *genitalium* ST159 clone, most likely circulating in MSM from the Paris, France, area.
